# Impact of Myocardial Fibrosis on Cardiovascular Structure, Function and Functional Status in Heart Failure with Preserved Ejection Fraction

**DOI:** 10.1007/s12265-022-10264-7

**Published:** 2022-07-05

**Authors:** Gavin A. Lewis, Anna Rosala-Hallas, Susanna Dodd, Erik B. Schelbert, Simon G. Williams, Colin Cunnington, Theresa McDonagh, Christopher A. Miller

**Affiliations:** 1 Division of Cardiovascular Sciences, School of Medical Sciences, Faculty of Biology, Medicine and Health, Manchester Academic Health Science Centre, University of Manchester, Oxford Road, Manchester, M13 9PL UK; 2 Manchester University NHS Foundation Trust, Southmoor Road, Wythenshawe, Manchester, M23 9LT UK; 3 Liverpool Clinical Trials Centre, Clinical Directorate, Faculty of Health and Life Sciences, University of Liverpool (a member of Liverpool Health Partners), Alder Hey Children’s NHS Foundation Trust, Liverpool, L12 2AP UK; 4 Department of Health Data Sciences, Institute of Population Health, Faculty of Health and Life Sciences, University of Liverpool (a member of Liverpool Health Partners), Block F, Waterhouse Bld, 1-5 Brownlow Street, Liverpool, L69 3GL UK; 5 Department of Medicine, University of Pittsburgh School of Medicine, Pittsburgh, PA USA; 6 UPMC Cardiovascular Magnetic Resonance Center, Heart and Vascular Institute, Pittsburgh, PA USA; 7 Clinical and Translational Science Institute, University of Pittsburgh, Pittsburgh, PA USA; 8 King’s College Hospital, Denmark Hill, London, SE5 9RS UK; 9 Wellcome Centre for Cell-Matrix Research, Division of Cell-Matrix Biology & Regenerative Medicine, School of Biology, Faculty of Biology, Medicine & Health, Manchester Academic Health Science Centre, University of Manchester, Oxford Road, Manchester, M13 9PT UK

**Keywords:** Heart failure with preserved ejection fraction, Myocardial fibrosis, Magnetic resonance imaging (MRI), Mediation analysis

## Abstract

Myocardial fibrosis, measured using cardiovascular magnetic resonance extracellular volume (ECV), is associated with adverse outcome in heart failure with preserved ejection fraction, but the mechanisms by which myocardial fibrosis exerts this deleterious effect are unclear. We performed mediation analyses of data from the Pirfenidone in Patients with Heart Failure and Preserved Left Ventricular Ejection Fraction (PIROUETTE) trial to determine whether myocardial fibrotic regression causes changes in cardiovascular function and functional status following antifibrotic therapy. Regression of myocardial fibrosis correlated with improvements in 6-min walk test and KCCQ clinical summary score. The only outcome variable that demonstrated a treatment effect was an increase in left ventricular ejection fraction (LVEF). The estimated average causal mediation effects of myocardial ECV, absolute myocardial extracellular matrix volume and absolute myocardial cellular volume on LVEF were 6.1%, 21.5% and 13.7%, respectively, none of which was significant and therefore not mediated by myocardial fibrosis. (PIROUETTE; NCT02932566).

**Graphical Abstract:**

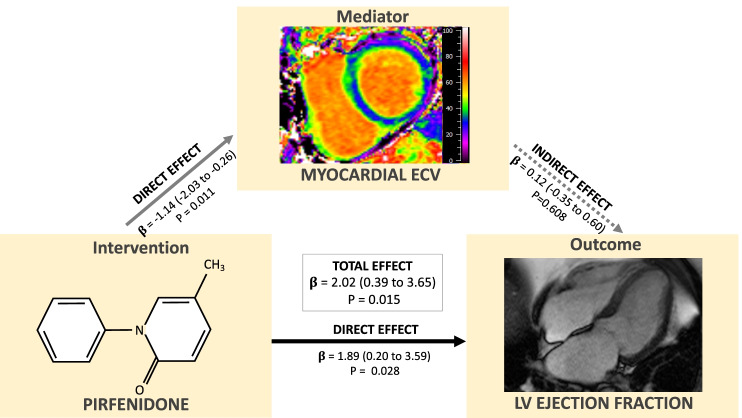

**Supplementary Information:**

The online version contains supplementary material available at 10.1007/s12265-022-10264-7.

## Introduction

Myocardial fibrosis, measured using cardiovascular magnetic resonance (CMR) extracellular volume (ECV), is associated with adverse outcome in patients with heart failure with preserved ejection fraction (HFpEF), including hospitalisation for heart failure (HF) and death [1–5].

The mechanisms by which myocardial fibrosis exerts this deleterious effect in HFpEF are unclear, but observational studies have demonstrated associations between myocardial fibrosis and myocardial stiffness, diastolic dysfunction, microvascular dysfunction and pulmonary hypertension [6–11].

The Pirfenidone in Patients with Heart Failure and Preserved Left Ventricular Ejection Fraction (PIROUETTE) study was a phase II, double-blind, placebo-controlled, randomised trial designed to evaluate the efficacy and mechanism of the novel antifibrotic agent, pirfenidone, in patients with HFpEF and myocardial fibrosis [12]. Pirfenidone is an orally bioavailable, small molecule antifibrotic agent that inhibits cardiac fibroblast synthesis and secretion of TGF-β1, proliferation and activation of fibroblasts and profibrotic pathways. Uniquely among cardiovascular interventions with antifibrotic effects that have been trialled in HFpEF, pirfenidone does not have a haemodynamic effect and, thus, in this regard, is a specific antifibrotic. As part of the trial protocol, participants underwent deep phenotyping, including detailed assessment of cardiovascular structure and function, circulating biomarkers and functional status.

Mediation analysis, conducted as part of a randomised controlled trial, allows estimation of the direct and indirect (via a mediator variable) effects of an intervention on outcome and thus can be used to determine whether the proposed pathophysiological mechanisms have causal effects [13].

The interventional nature of the PIROUETTE trial and the specific antifibrotic nature of pirfenidone, in conjunction with mediation analysis, provide novel opportunity to investigate the causal impact of myocardial fibrosis on cardiovascular structure and function, circulating biomarkers and functional status, which has the potential to help understand why myocardial fibrosis is associated with adverse outcome.

This study aimed to determine whether myocardial fibrosis causes changes in cardiovascular structure and function, circulating biomarkers and functional status, by conducting a mediation analysis of data from the PIROUETTE trial.

## Methods

### Study Design and Patient Selection

The trial design and results of the PIROUETTE trial (Clinicaltrials.gov NCT02932566) have been published previously [12, 14]. In brief, between March 7, 2017, and December 19, 2018, 94 patients with HFpEF and myocardial fibrosis were randomised to receive pirfenidone or placebo treatment for 52 weeks. Eligibility requirements included patients being ≥ 40 years of age, symptoms and signs of heart failure, left ventricular ejection fraction of ≥ 45% and elevated natriuretic peptides (brain natriuretic peptide (BNP) ≥ 100 pg/ml or N-terminal pro-B-type natriuretic peptide (NT-proBNP) ≥ 300 pg/ml or BNP ≥ 300 pg/ml or NT-proBNP ≥ 900 pg/ml if atrial fibrillation (AF) present). Patients deemed eligible underwent CMR, and those with evidence of myocardial fibrosis, defined as having an ECV of 27% or higher, were randomised to treatment with either pirfenidone or matching placebo for 52 weeks stratified by sex. Exclusion criteria included patients having an alternative cause of symptoms such as significant respiratory disease, obesity or anaemia; hypertrophic cardiomyopathy, pericardial constriction or infiltrative cardiomyopathy; and contraindication to CMR imaging. The primary outcome was change in myocardial fibrosis from baseline to 52 weeks, measured using CMR ECV.

The trial was sponsored by Manchester University NHS Foundation Trust, and trial management, independent data management and independent statistical analyses were performed by Liverpool Clinical Trials Centre, a UK Clinical Research Collaboration Clinical Trials Unit. Trial conduct was overseen by a trial steering committee. The study protocol was approved by a research ethics committee. Patients were identified at six UK hospitals and study visits took place at Manchester University NHS Foundation Trust. All patients provided written informed consent.

### Study Procedures and Analysis

The trial procedures, analysis methods and outcome measurements have been described previously [12, 14]. In brief, echocardiography, CMR, electrocardiography, 6-min walk test, laboratory tests and the Kansas City Cardiomyopathy Questionnaire (KCCQ) were performed both at baseline and repeated after 52 weeks of treatment. ^31^Phosphorous magnetic resonance spectroscopy (^31^P-MRS) was also performed at baseline and 52 weeks in a subset of patients as part of a predefined sub-study (*n* = 60).

Myocardial ECV was calculated from basal and mid-left ventricular (LV) short axis pre- and post-contrast T1 maps (Modified Look-Locker Inversion [MOLLI] recovery). Images were acquired before and 15 min after administration of gadolinium contrast (0.15 mmol/kg of gadoterate meglumine), as ECV = (1 – haematocrit) × [ΔR1_myocardium_]/[ΔR1_bloodpool_], where ΔR1 is the difference in relaxation rates (1/T1) between the pre- and post-contrast [4]. The haematocrit was measured on the same day as the CMR scan. Absolute myocardial extracellular matrix (ECM) volume was calculated as the product of LV myocardial volume (LV mass divided by the known specific gravity of myocardium [1.05 g/ml]) and ECV. Absolute myocardial cellular volume was calculated as the product of LV myocardial volume (LV mass divided by the known specific gravity of myocardium [1.05 g/ml]) and (1 – ECV). Full details can be found in the trial protocol paper [14].

### Statistical Analysis

Analysis was conducted on an intention to treat basis, including all randomised patients retained in their randomised treatment groups. Continuous data are presented as mean ± standard deviation (SD) or as median (interquartile range (IQR)), as appropriate. Categorical data are presented as counts and percentages. Correlation analyses were used to assess associations between change in ECV (week 52 value minus baseline value) and change in selected secondary outcome variables that reflected cardiac mechanical and electrical function, circulating biomarkers and functional status. Pearson’s or Spearman’s correlation coefficients were used as appropriate following Shapiro–Wilk testing for normality. Analyses were performed in Stata (version 14.0, StataCorp, College Station, TX) and SAS (version 9.4, SAS Institute, Inc., Cary, NC).

### Mediation Analysis

**Fig. 1:**
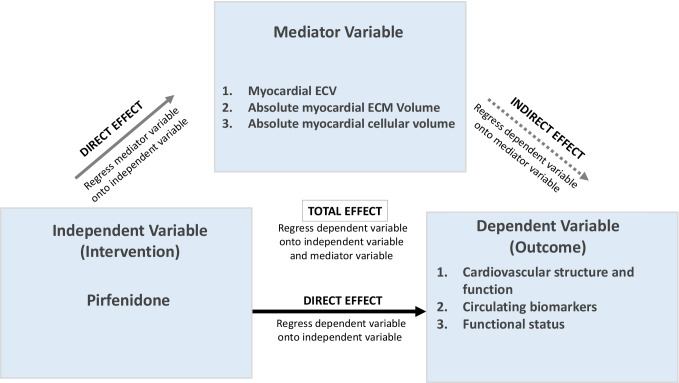
Mediation analysis method. Mediation analysis as described by Baron and Kenny [15]. To proceed onto mediation analysis, the independent variable must have a significant effect on both the mediator variable and the outcome variable (direct effect). If either relationship is non-significant, this indicates non-mediation. If significant, the outcome variable is regressed onto both the independent and mediator variables; if the independent variable no longer effects the outcome variable, this indicates full mediation; if the independent variable still predicts the outcome variable but with a smaller size, this indicates partial mediation

Mediation analyses were conducted in order to determine whether changes in myocardial fibrosis, measured using ECV and absolute ECM volume, and changes in myocardial cellular volume (the mediator variables) following antifibrotic therapy, caused changes in cardiovascular structure and function, circulating biomarkers and functional status (the outcome variables) (Fig. 1).

Mediation analyses were conducted using the Baron and Kenny approach [15], under a structural equation modelling (SEM) framework (Stata package medsem), in order to estimate the average causal mediation effect (ACME) of each mediator, adjusting for confounders (baseline covariates that predicted both the mediator and outcome at the 10% significance level). For each potential outcome, the analysis was only performed if both of the following conditions were satisfied:

1. The antifibrotic therapy had a significant effect on the outcome at the 5% level (i.e. *p* < 0.05 for the treatment effect in an analysis of covariance (ANCOVA) model of the outcome, adjusting for treatment, sex and baseline value of the outcome variable).
2. The antifibrotic therapy had a significant effect on the mediator variables (i.e. ECV, absolute ECM volume and myocardial cellular volume) at the 10% level (i.e. *p* < 0.1 for the treatment effect in the ANCOVA models of ECV, absolute ECM volume and myocardial cellular volume, adjusting for treatment, sex and baseline values of the mediator variables).

The outcome variables for this study were all secondary outcome measurements in the main PIROUETTE trial. The outcome variables reflect cardiovascular structure and function, circulating biomarkers and functional status. They were selected for use in this study because they are variables that, based on the published literature and clinical judgement, are associated with adverse outcome in HFpEF or were hypothesised to be impacted by myocardial fibrosis. The selection of the outcome variables was prospective; i.e. outcome variables were selected before data lock for the trial had occurred, thus before the results of the trial were known and were prespecified in an ‘Additional Statistical Analysis Plan’ that was written before data lock.

## Results

### Patients

Baseline characteristics of the 94 patients that were randomised are presented in Table 1. The mean age of patients was 78 years, and 46% were female. Nearly all patients had New York Heart Association functional class II or III symptoms (95%), the mean LV ejection fraction was 64%, and the median NT-proBNP was 1104 pg/ml. The mean myocardial ECV was 30.1%. At the end of the trial, 12 patients had withdrawn from the study and two had died. No patient was lost to follow-up. Only patients with complete data were included in the mediation analysis (*n* = 79).

**Table 1:** Baseline characteristics

Characteristic	Patients (*N* = 94)
Age, years	78 ± 7.4
Female, no. (%)	43 (46)
White race, no. (%)	88 (94)
Hypertension, no. (%)	79 (84)
Diabetes, no. (%)	28 (30)
Systolic blood pressure, mmHg	136 ± 22.5
Diastolic blood pressure, mmHg	76 ± 15.3
BMI, kg/m^2^	31 ± 5.6
eGFR, mls/min	57 ± 16.7
Haemoglobin, g/dL	12.9 ± 1.5
Log NT-proBNP, pg/ml	7.0 ± 0.9
HS-Troponin T, pg/ml	28.1 ± 31.0
QRS duration, ms	105 ± 16.8
Myocardial ECV, %	30.1 ± 2.7
Absolute myocardial ECM volume, ml	36.7 ± 11.4
Absolute myocardial cellular volume, ml	85.1 ± 24.1
Left ventricular end-diastolic volume index, ml/m^2^	63 ± 36.3
Left ventricular ejection fraction, %	64 ± 23.3
Left ventricular mass index, g/m^2^	65 ± 15.4
Average e′, cm/s	8.8 ± 2.5
Average E/e′, cm/s	12.2 ± 3.4
Global longitudinal strain, %	− 16.0 ± 3.5
Torsion, degrees/cm	1.5 ± 0.7
PCr:ATP ratio	1.3 ± 0.4
Right ventricular end-diastolic volume index, ml/m^2^	69 ± 16.4
Right ventricular ejection fraction, %	52 ± 9.5
Pulmonary artery systolic pressure, mmHg	33 ± 12.8
Left atrial volume index, ml/m^2^	70.4 ± 18.6
Left atrial strain (reservoir), %	16.9 ± 7.6
Left atrial strain (booster), %	12.4 ± 4.3
Left atrial strain (conduit), %	10.4 ± 3.8
Aortic distensibility, 10^−3^/mmHg	1.6 ± 0.9
Pulse-wave velocity, m/s	12.6 ± 5.0
6-min walk test, m	265 ± 114.6
KCCQ clinical summary score	56.0 ± 19.9

Values are presented as mean ± SD unless stated

*ATP*, adenosine triphosphate; *BMI*, body mass index; *ECM*, extracellular matrix; *ECV*, extracellular matrix volume; *eGFR*, estimated glomerular filtration rate; *HS-Troponin T*, high-sensitivity troponin T; *KCCQ*, Kansas City Cardiomyopathy Questionnaire; *NT-proBNP*, n-terminal pro B-type natriuretic peptide; *PCr*, phosphocreatine

### Associations with Change in ECV

**Fig. 2:**
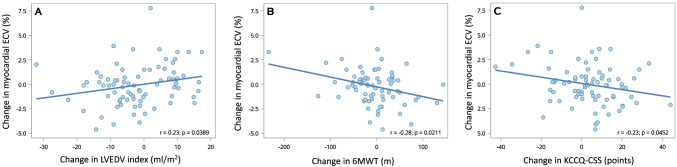
Associations with change in extracellular volume (ECV) from baseline to week 52. Correlations between change in ECV and **A** change in left ventricular end-diastolic volume (LVEDV) index, **B** change in 6-min walk test (6MWT) and **C** change in Kansas City cardiomyopathy questionnaire Clinical Summary Score (KCCQ-CSS)

Change in myocardial ECV from baseline to week 52 showed a positive correlation with change in left ventricular end-diastolic volume indexed for body surface area (*r* = 0.23, *p* = 0.039), and inverse correlations with 6-min walk test distance (*r* =  − 0.28, *p* = 0.021), and KCCQ Clinical Summary Score (*r* =  − 0.23, *p* = 0.045) (Fig. 2 and Table S1 in Supplementary Appendix), although all the associations were weak.

### Mediation Analysis

Pirfenidone had a significant effect on the mediator variables measuring myocardial fibrosis (myocardial ECV and absolute myocardial ECM volume). The effect of pirfenidone on the mediator variable myocardial cellular volume was not significant at the conventional 5% level but was significant at the 10% level, and thus, as prespecified, this mediator variable was also included in the mediation analysis (Table 2).

**Table 2:** Treatment effect on each mediator variable

Mediator variable	Estimated 95% CI	*P* value associated with treatment effect
Myocardial ECV, %	− 1.21 (− 2.12 to − 0.31)	0.009
Absolute myocardial ECM volume, ml	− 3.06 (− 4.96 to − 1.16)	0.002
Absolute myocardial cellular volume, ml	− 3.41 (− 7.28 to 0.47)	0.084

Analysis of covariance models, adjusted for baseline value of the mediator variable, sex and treatment group. *CI*, confidence interval; *ECM*, extracellular matrix; *ECV*, extracellular matrix volume

The only outcome variable that demonstrated a treatment effect was LV ejection fraction (*p* = 0.011) (Table 3).

**Table 3:** Treatment effect on each outcome variable

Outcome variable	*P* value associated with treatment effect
Systolic blood pressure, mmHg	0.72
Diastolic blood pressure, mmHg	0.49
BMI, kg/m^2^	0.31
eGFR, ml/min	0.13
Haemoglobin, g/dL	0.49
Log NT-proBNP, pg/ml	0.079
HS-Troponin T, pg/ml	0.64
QRS duration, ms	0.93
LV end-diastolic volume index, ml/m^2^	0.80
LV ejection fraction, %	0.011
LV mass index, g/m^2^	0.10
Average e′, cm/s	0.73
Average E/e′, cm/s	0.76
Global longitudinal strain, %	0.10
Torsion, degrees/cm	0.55
PCr:ATP	0.62
RV end-diastolic volume index, ml/m^2^	0.58
RV ejection fraction, %	0.27
Pulmonary artery systolic pressure, mmHg	0.89
LA volume index, ml/m^2^	0.83
LA strain (reservoir), %	0.78
LA strain (booster), %	0.75
LA strain (conduit), %	0.50
Aortic distensibility, 10^−3^/mmHg	0.08
Pulse wave velocity, m/s	0.80
6-min walk test, m	0.22
KCCQ Clinical Summary Score	0.09

Analysis of covariance models, adjusted for baseline value of the outcome variable, sex and treatment group. *ATP*, adenosine triphosphate; *BMI*, body mass index; *eGFR*, estimated glomerular filtration rate; *HS-Troponin T*, high-sensitivity troponin T; *KCCQ*, Kansas City Cardiomyopathy Questionnaire; *LA*, left atrial; *LV*, left ventricular; *NT-proBNP*, N-terminal pro B-type natriuretic peptide; *PCr*, phosphocreatine; *RV*, right ventricular

No baseline covariates were found to predict the mediator variables and LV ejection fraction; thus, no baseline covariates were required to be included in the mediation analysis (Table S2 in Supplementary Appendix).

**Fig. 3:**
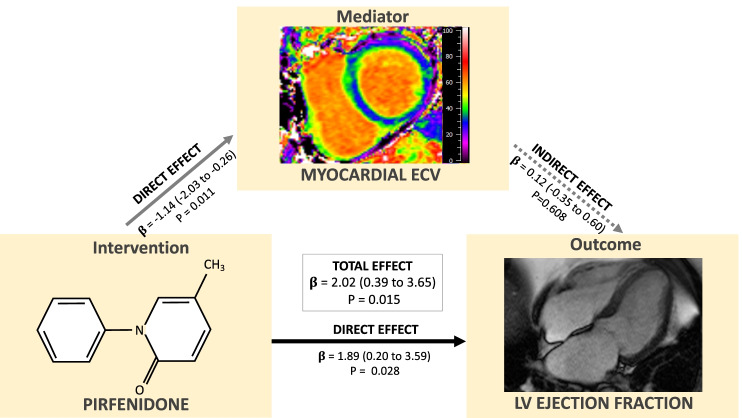
Mediation model. Illustration of the mediation model. Pirfenidone (treatment intervention) has a direct effect on left ventricular (LV) ejection fraction (outcome variable) and a direct effect on extracellular volume (ECV) (mediator variable). Pirfenidone also has an indirect effect on LV ejection fraction via the effect on ECV. Mediation analysis determines whether the change in the mediator variable (ECV) causes the change in the outcome variable (LV ejection fraction), by calculating the proportion of the total effect of treatment on the outcome variable that is due to the indirect effect of treatment on the outcome variable acting via the mediator (ECV), i.e. the proportion of the total treatment effect that is mediated via the mediation variable (ECV). As per Table 4, the proportion of the total effect of pirfenidone on LV ejection fraction that was mediated by ECV was 6.1%, which was not significant (*p* = 0.608)

In the mediation analysis, the estimated average causal mediation effects of myocardial ECV, absolute myocardial ECM volume and absolute myocardial cellular volume on LV ejection fraction were 6.1%, 21.5% and 13.7%, respectively, none of which was significant (*p* = 0.608, *p* = 0.123 and *p* = 0.186, respectively) (Table 4 and Fig. 3).

**Table 4:** Mediation analysis

Mediator	Direct effect of treatment on week 52 LVEF (%)	Direct effect of treatment on week 52 mediator	Indirect effect of treatment on week 52 LVEF (%)	Total effect of treatment on week 52 LVEF (%)	Proportion of effect mediated (%)
Myocardial ECV, %	1.89 (0.20 to 3.59); *p* = 0.028	− 1.14 (− 2.03 to − 0.26); *p* = 0.011	0.12 (− 0.35 to 0.60); *p* = 0.608	2.02 (0.39 to 3.65); *p* = 0.015	6.1; *p* = 0.608
Absolute myocardial ECM volume, ml	1.88 (0.22 to 3.53); *p* = 0.026	− 3.13 (− 4.99 to − 1.28); *p* = 0.001	0.51 (− 0.14 to 1.17); *p* = 0.123	2.39 (0.81 to 3.97); *p* = 0.003	21.5; *p* = 0.123
Absolute myocardial cellular volume, ml	1.93 (0.38 to 3.48); *p* = 0.015	− 3.83 (− 7.56 to − 0.10); *p* = 0.044	0.30 (− 0.15 to 0.76); *p* = 0.186	2.23 (0.70 to 3.78); *p* = 0.004	13.7; *p* = 0.186

Values are mean (95% confidence interval). *ECM*, extracellular matrix; *ECV*, extracellular matrix volume; *LVEF*, left ventricular ejection fraction

## Discussion

Over the past decade, observational data have demonstrated non-infarct myocardial fibrosis to be strongly associated with adverse prognosis across a range of cardiovascular conditions, including HFpEF. As such, the myocardial interstitium has emerged as a potential therapeutic target; indeed, this was the focus of the PIROUETTE trial, which targeted patients with HFpEF and evidence of myocardial fibrosis with a specific antifibrotic intervention. Nevertheless, whilst observational data are useful, the observed association between myocardial fibrosis and adverse cardiovascular outcomes does not establish causality. Randomised controlled trials provide an opportunity to do this.

The PIROUETTE trial established the efficacy of pirfenidone, an antifibrotic without blood pressure effect, to attenuate myocardial fibrosis in HFpEF. The associated reduction in natriuretic peptide levels over time provides support for the extracellular matrix having a causal role in HFpEF and being an efficacious therapeutic target. The deep phenotyping conducted as part of the PIROUETTE trial, in conjunction with the described mediation analysis, potentially provided an opportunity to determine the causal impact myocardial fibrosis has on other aspects of cardiovascular structure and function, such as myocardial contractile and electrical and energetic function, as well as other factors such as functional status.

Unfortunately, the only secondary outcome of the trial prospectively selected as an outcome for this study that demonstrated a significant change from baseline to 52 weeks in response to pirfenidone, in comparison to placebo, was LV ejection fraction. There were notable trends towards improvement in other variables, such as KCCQ Clinical Summary Score (*p* = 0.09) and global longitudinal strain (*p* = 0.10), but none was significant, possibly reflecting lack of power. Indeed, the sample size for the PIROUETTE trial was calculated based on the primary outcome; the trial was not powered for secondary outcomes.

Despite being statistically significant, the magnitude of change in LV ejection fraction associated with treatment with pirfenidone was small (between-group difference, 2.16%; 95% confidence interval [CI], 0.51 to 3.81), and the clinical relevance is unclear. It is perhaps unsurprising, therefore, that regression of myocardial fibrosis was not found to mediate this effect.

Regression of myocardial fibrosis did correlate with improvements in functional status, such as 6-min walk test distance and KCCQ clinical summary score. Whilst the associations were relatively weak, these findings are novel and provide more support for myocardial fibrosis having an important mechanistic role in HFpEF.

### Limitations

As discussed, the PIROUETTE trial was not powered for secondary outcomes; thus, the findings of this study are considered exploratory. The analyses conducted as part of the current study were not included in the Statistical Analysis Plan for PIROUETTE and thus are considered post hoc. Nevertheless, the analyses conducted in this study were prespecified in an ‘Additional Statistical Analysis Plan’ that was written before trial data lock. As discussed, the selection of outcome variables to include in this study was prospective and performed before data lock. Finally, no adjustment for multiple comparisons was performed; therefore, false positive results cannot be excluded.

## Conclusion

In this analysis of the PIROUETTE trial, regression of myocardial fibrosis was associated with improvements in functional status. The small improvement in left ventricular ejection fraction associated with pirfenidone was not mediated by myocardial fibrosis regression.

## Supplementary Information

Below is the link to the electronic supplementary material.

Supplementary file1 (DOCX 43 KB)

## Data Availability

Anonymised data will be made available in full by reasonable request in writing to the corresponding author following appropriate completion of a data sharing agreement.

## Abbreviations

^31^P-MRS: 31Phosphorous cardiac magnetic resonance spectroscopy
ANCOVA: Analysis of covariance
BNP: Brain natriuretic peptide
CMR: Cardiac magnetic resonance
ECV: Extracellular volume
HF: Heart failure
HFpEF: Heart failure with preserved ejection fraction
KCCQ: Kansas City Cardiomyopathy Questionnaire
LVEF: Left ventricular ejection fraction
NT-proBNP: N-terminal pro B-type natriuretic peptide
