# Supplementary material for: Impact of Myocardial Fibrosis on Cardiovascular Structure, Function and Functional Status in Heart Failure with Preserved Ejection Fraction
Source: J Cardiovasc Transl Res. 2022 Jul 5;15(6):1436–43. doi: 10.1007/s12265-022-10264-7 (PMC9722869; doi:10.1007/s12265-022-10264-7)
Supplement: Supplementary file 1 — Supplementary file1 (DOCX 43 KB) [file 12265_2022_10264_MOESM1_ESM.docx]

**Impact of myocardial fibrosis on cardiovascular structure, function and functional status in heart failure with preserved ejection fraction**

**SUPPEMENTARY APPENDIX**

Table of Contents

[Supplementary Table S1. Associations with change in ECV from baseline to week 52 2](#_Toc54121863)

[Supplementary Table S2. Impact of baseline covariates on the mediator variables and LV ejection fraction 3](#_Toc54121864)

## Supplementary Table S1. Associations with change in ECV from baseline to week 52

| **Variable** | **Change at 52-weeks** | **Correlation coefficient*** | **P-value** |
| --- | --- | --- | --- |
| Systolic blood pressure - mmHg | -1.0 ± 22.9 | 0.01 | 0.91 |
| Diastolic blood pressure - mmHg | -0.9 ± 16.5 | -0.20* | 0.07 |
| BMI - kg/m2 | -0.4 ± 1.5 | 0.00* | 0.97 |
| eGFR - ml/min | -2.3 ± 11.2 | -0.05 | 0.69 |
| Haemoglobin - g/dL | -0.3 ± 1.0 | -0.54 | <0.001 |
| Log NT-proBNP - pg/ml | -0.1 ± 0.7 | 0.15* | 0.19 |
| HS-Troponin T - pg/ml | -1.5 ± 10.7 | -0.17* | 0.14 |
| QRS duration - ms | -0.9 ± 6.4 | -0.10 | 0.37 |
| LV end diastolic volume index - ml/m2 | -1.9 ± 10.2 | 0.23 | 0.039 |
| LV ejection fraction - % | 0.4 ± 4.0 | 0.05* | 0.65 |
| LV mass index - g/m2 | -1.3 ± 7.2 | 0.05* | 0.63 |
| Average e’ - cm/s | -0.9 ± 2.2 | -0.04* | 0.70 |
| Average E/e’ - cm/s | 0.6 ± 3.8 | 0.11* | 0.33 |
| Global Longitudinal Strain - % | -0.3 ± 3.5 | -0.07 | 0.56 |
| Torsion - degrees/cm | 0.1 ± 1.0 | -0.12 | 0.29 |
| PCr:ATP | 0.1 ± 0.5 | -0.19 | 0.19 |
| RV end diastolic volume index - ml/m2 | 1.3 ± 11.3 | 0.14 | 0.22 |
| RV ejection fraction - % | 0.1 ± 7.1 | -0.02* | 0.84 |
| Pulmonary artery systolic pressure - mmHg | 0.4 ± 11.3 | 0.08* | 0.60 |
| LA volume index - ml/m2 | 2.5 ± 12.7 | 0.18* | 0.10 |
| LA strain (reservoir) - % | 0.4 ± 5.6 | 0.04 | 0.73 |
| LA strain (booster) - % | 1.7 ± 4.5 | -0.05 | 0.77 |
| LA strain (conduit) - % | -0.3 ± 3.9 | 0.13* | 0.24 |
| Aortic distensibility – 10^-3^/mmHg | -0.2 ± 0.9 | -0.04* | 0.75 |
| Pulse Wave Velocity - m/s | 2.3 ± 6.6 | -0.02* | 0.89 |
| 6-minute walk test - m | -5.3 ± 56.5 | -0.28 | 0.021 |
| KCCQ Clinical Summary Score | 3.6 ± 14.9 | -0.23 | 0.045 |

Table 2 Legend. Values are mean ± SD. *Spearman’s correlation used rather than Pearson’s due to P<0.05 in Shapiro-Wilk test for normality. ATP – adenosine triphosphate; BMI – body mass index; ECM – extracellular matrix; ECV – extracellular matrix volume; eGFR – estimated glomerular filtration rate; HS-Troponin T – high-sensitivity troponin T; KCCQ – Kansas City Cardiomyopathy Questionnaire; LA – left atrial; LV – left ventricular; NT-proBNP – n-terminal pro B-type natriuretic peptide; PCr – phosphocreatine; RV – right ventricular.

## Supplementary Table S2. Impact of baseline covariates on the mediator variables and LV ejection fraction

| **Baseline variable** | **P-values for Outcome Model:**  *Week 52 LV ejection fraction (%)* | **P-values for Mediation Models:** | | |
| --- | --- | --- | --- | --- |
|  |  | *Week 52 Myocardial ECV (%)* | *Week 52 Absolute Myocardial ECM volume (ml)* | *Week 52 Absolute Myocardial cell volume (ml)* |
| Systolic blood Pressure – mmHg | 0.09 | 0.57 | 0.36 | 0.83 |
| Diastolic blood pressure – mmHg | 0.26 | 0.24 | 0.07 | 0.04 |
| BMI - kg/m2 | 0.27 | 0.40 | 0.81 | 0.66 |
| eGFR - ml/min | 0.88 | 0.74 | 0.01 | 0.03 |
| Haemoglobin - g/dL | 0.99 | 0.74 | 0.06 | 0.07 |
| Log NT-proBNP - pg/ml | 0.60 | 0.04 | 0.11 | 0.90 |
| HS-troponin T pg/ml | 0.59 | 0.03 | 0.03 | 0.33 |
| QRS duration – ms | 0.29 | 0.07 | 0.72 | 0.14 |
| LV end diastolic volume index - ml/m2 | 0.53 | 0.43 | 0.002 | 0.66 |
| LV ejection fraction - % | <0.001 | <0.001 | <0.001 | <0.001 |
| LV mass index - g/m2 | 0.25 | 0.12 | 0.006 | 0.98 |
| Average e’ - cm/s | 0.42 | 0.86 | 0.41 | 0.58 |
| Average E/e’ - cm/s | 0.34 | 0.33 | 0.40 | 0.52 |
| Global Longitudinal Strain - % | 0.70 | 0.04 | 0.46 | 0.80 |
| Torsion - degrees/cm | 0.85 | 0.50 | 0.88 | 0.53 |
| PCr:ATP | 0.13 | 0.64 | 0.33 | 0.40 |
| RV end diastolic volume index - ml/m^2^ | 0.09 | 0.63 | 0.24 | 0.81 |
| RV ejection fraction - % | 0.77 | 0.33 | 0.43 | 0.97 |
| Pulmonary artery systolic pressure - mmHg | 0.26 | 0.96 | 0.62 | 0.93 |
| LA volume index - ml/m2 | 0.33 | 0.21 | 0.39 | 0.74 |
| LA strain (reservoir) - % | 0.82 | 0.03 | 0.01 | 0.24 |
| LA strain (booster) - % | 0.88 | 0.14 | 0.17 | 0.48 |
| LA strain (conduit) - % | 0.60 | 0.06 | 0.11 | 0.20 |
| Aortic distensibility - 10^-3^/mmHg | 0.76 | 0.87 | 0.92 | 0.74 |
| Pulse Wave Velocity - m/s | 0.87 | 0.13 | 0.27 | 0.07 |
| 6-minute walk test - m | 0.21 | 0.76 | 0.68 | 0.54 |
| KCCQ Clinical Summary Score | 0.55 | 0.48 | 0.51 | 0.90 |

Table S2 Legend. Analysis of covariance models, adjusted for baseline values of mediator or outcome variables, sex and treatment group. ATP – adenosine triphosphate; BMI – body mass index; eGFR – estimated glomerular filtration rate; HS-Troponin T – high-sensitivity troponin T; KCCQ – Kansas City Cardiomyopathy Questionnaire; LA – left atrial; LV – left ventricular; NT-proBNP – N-terminal pro B-type natriuretic peptide; PCr – phosphocreatine; RV – right ventricular.
